# Maternal Depression and Sleep Problems in Early Childhood: A Meta-Analysis

**DOI:** 10.1007/s10578-024-01717-y

**Published:** 2024-06-05

**Authors:** Owen Orton, Ayten Bilgin

**Affiliations:** 1 Department of Psychology, University of Essex, Colchester, UK; 2 Folkestone, UK

**Keywords:** Prenatal depression, Postnatal depression, Infant sleep problems, Meta-analysis

## Abstract

Both prenatal and postnatal maternal depression have been associated with increased sleep problems in early childhood. However, this association is less consistent for postnatal depression, and the strength of the association remains unclear. The aim of the current study was to provide a quantitative synthesis of the literature to estimate the magnitude of the association between maternal depression and sleep problems in early childhood. Medline, PsycINFO, PsycARTICLES, Web of Science, and Scopus were searched for prospective longitudinal studies from 1970 to December 2022. Of 117 articles screened, 22 studies met the inclusion criteria. Both prenatal depression (OR = 1.82; 95% CI = 1.28–2.61) and postnatal depression (OR = 1.65; 95% CI = 1.50–1.82) were associated with increased likelihood of sleep problems in early childhood. The heterogeneity between the studies was significant and high both for prenatal (*Q* = 432.323; *I*^2^ = 97.456, *P* < .001) and postnatal depression (*Q* = 44.902, *I*^2^ = 65.594, *P* < .001), which mean that conclusions are tentative and need to be considered within the possible influence of unmeasured confounding. However, mitigating depression symptoms in mothers both during pregnancy and in the postnatal period would be an effective strategy for reducing sleep problems in children.

## Introduction

The period from birth to 3 years of age constitutes the most critical years for healthy brain development [19], and provides the foundation for future life achievement [12]. To ensure healthy growth, human infants need to have healthy sleeping patterns during this period [27]. However, approximately 20% to 35% of children show symptoms of sleep problems during the first 3 years such as short sleep duration, frequent night wakings, and difficulties in settling into sleep, which are one of the biggest sources of concern for parents in the early years [15, 52, 62]. Sleep problems in children are associated with increased levels of stress and fatigue in parents which could influence their sensitive parenting, as well as decrease their productivity at work [7]. Further, there is evidence that sleep problems that are established during the first 3 years of life can persist throughout childhood [15, 63, 71], and are associated with a range of outcomes including cognitive difficulties such as problems in nonverbal reasoning [9], and language skills [22], child-parent attachment difficulties [10, 64], as well as internalizing and externalizing problems lasting into adolescence and adulthood [61, 67].

Given its potential negative impact on the parents and the children, there has been an interest to understand the factors which might contribute to the development of sleep problems in children. Sadeh and Anders’ [60] transactional model proposed that ongoing bidirectional associations between intrinsic (e.g., temperament, medical) and extrinsic (e.g., parental soothing behaviors, depression, culture) factors explain the development of sleep problems. Among these factors, it was suggested that maternal depression plays a key role in the development of sleep problems in early childhood [3, 26, 68, 73, 74]. Maternal depressive symptoms could impact sleep problems in early childhood through several mechanisms. In the prenatal period, the association between maternal depression and sleep problems in early childhood has been explained by the prenatal programming that adversely influences fetal development [29]. It was shown that depressive symptoms during pregnancy could expose the fetus to high levels of maternal glucocorticoids and pro-inflammatory cytokines, and program an adverse offspring phenotype which could explain why maternal depressive symptoms during pregnancy predicts increased risk of sleep problems in children [16, 43, 54].

In the postnatal period, it was suggested that maternal depression could influence sleep problems in early childhood due to interfering with maternal bedtime and nighttime behaviors [62]. There is evidence that mothers with depressive symptoms are more likely to be present in the same room with their infant and spend higher amount of time in close physical contact with their infants during the night due to thoughts of helplessness/loss of control and perception of themselves as inadequate parents [68]. This is likely to interfere with children’s ability to acquire self-soothing skills which are required to sleep through the night or to self-soothe back to sleep once awoken [62]. Further, maternal depression and early childhood sleep problems may be associated due to shared genetic vulnerability [72].

Although the research evidence is consistent for the association between prenatal depression and sleep problems in early childhood [26, 45, 47, 51, 66, 69], the effect sizes vary across different studies, and it is unclear how large the effect of prenatal depressive symptoms is on sleep problems in early childhood. Further, the evidence regarding the link between postnatal maternal depression and sleep in early childhood is mixed with some studies revealing nonsignificant associations [17, 56]. The nonsignificant associations could be due to the assessment time of maternal depression as one of these studies assessed maternal depression at delivery [17] and the other assessed it concurrently and as an outcome of sleep problems in children [56].

There are several potential factors which could moderate the association between maternal depression and sleep problems in early childhood. First, both the age at assessment of postnatal maternal depression and child sleep problems could moderate the association between maternal depression and sleep problems in early childhood. Regarding the assessment of postnatal maternal depression, it was suggested that earlier exposure to maternal depression has a higher impact than later exposure [31]. Since the first 6 months of after birth is a particularly important period for the development of healthy sleep patterns in children [27, 60], maternal regulation of infant nighttime behavior may be most needed in the first 6 months of life [62]. The presence of depressive symptoms during the first 6 months of life might limit mothers’ capacity to provide this guidance. Regarding the age at assessment of sleep problems in early childhood, there is evidence that sleep problems arising before the first year of life is more likely to persist throughout early childhood [15]. Thus, it is important to understand whether the impact of maternal depression on child sleep problems depends on whether sleep problems were assessed before or after the first year.

Second, maternal age could be a moderator on the association between maternal depressive symptoms and child sleep problems. Younger mothers might have less economic and social resources and live in less supportive and less stable households than older mothers [25]. Further, there is evidence that younger mothers are more likely to have symptoms of postpartum depression and their children are more likely to have emotional and behavioral problems [1]. Thus, younger mothers might have less resources to mitigate the impact of depression which may influence their abilities to set bed-time routines and manage infant sleep effectively [14].

Third, the association between maternal depression and sleep problems in childhood could depend on the income level of the country. It was shown that the prevalence of maternal depressive symptoms both during the prenatal and postnatal periods is higher in many low and middle income countries than found in high-income countries [55], which might be due to factors such as higher life stress and domestic violence and low socioeconomic status [39]. Thus, the association between maternal depressive symptoms and sleep problems in early childhood might change depending on the income level of the countries.

Fourth, it is also important to note the potential influence of the type of assessment of sleep problems in children on the association between maternal depression and sleep problems in early childhood. The most common method for evaluating sleep problems in early childhood is parent’s subjective reports [44]. Although most studies use well-established parent reports to measure sleep problems in children, some studies only used a one-item question, which might influence the link between depressive symptoms and sleep problems in children.

The aim of the current study is to provide a quantitative synthesis of the literature to estimate the association between maternal depression and sleep problems in early childhood up to 3 years of age focusing on depression during prenatal and postnatal periods separately. To attain a clear understanding of the methodological factors that may amplify, or attenuate observed associations, sample, and methodological moderators (i.e., child age at assessment of sleep problems, maternal age, income level of the country, type of assessment of sleep problems in children, time of assessment of postnatal depression) will be examined to determine whether they predict between-study variation.

## Methods

### Research Design and Methods

This meta-analysis was registered with the PROSPERO International prospective register of systematic reviews with the following number: CRD42022383588, and was conducted in line with the Preferred Reporting Items for Systematic Reviews and Meta-Analyses guidelines [49]. The criteria for the search strategy, the selection of studies, data extraction and analyses were prespecified.

### Study Selection Criteria

Prospective longitudinal studies were eligible for this meta-analysis. Studies were included in the analysis according to 2 criteria. First, articles should contain reports on prenatal and/or postnatal maternal depression and infant and toddler sleep problems (e.g., short sleep duration, frequent night-waking, difficulties settling into sleep). Second, sleep problems in children should be measured up to 3 years of age given that the current study focuses on sleep problems in early childhood. Studies were excluded if mothers retrospectively reported on prenatal or postnatal depression given that retrospective reports may be subjected to recall bias. We further excluded randomized control trials, and cross-sectional studies since the current study aims to investigate the longitudinal associations between maternal depression and child sleep problems.

### Search Strategy

**Fig. 1:**
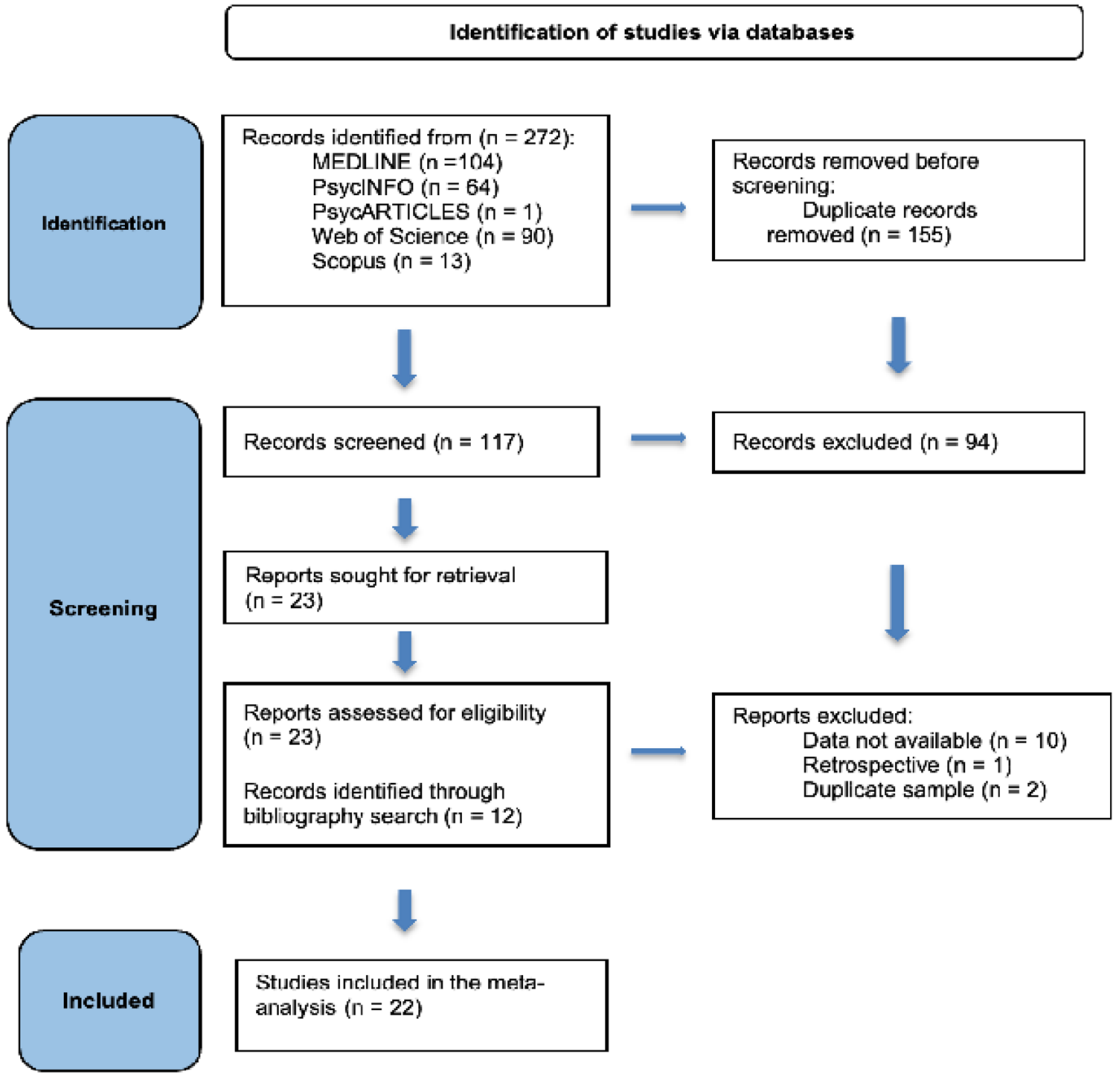
Search strategy

A literature search was conducted for prospective longitudinal studies of maternal depression and sleep problems in infancy, from 1970 up to December 2022. The article search was finalized on 12 December 2022. The following electronic databases were searched: Medline, PsycINFO, PsycARTICLES, Web of Science, and Scopus. The search strategy included the following search terms: ('prenatal depression' OR 'perinatal depression' OR 'postpartum depression' OR 'antenatal depression' OR 'maternal depress*' OR 'mother depress*) AND (‘infant sleep*’ OR sleep*). The Medline search yielded 104 articles, PsycINFO yielded 64 articles, PsycARTICLES yielded 1 article, Web of Science yielded 90 articles, and Scopus yielded 13 articles. Overall, 272 articles were included in the literature search. 155 duplicates were removed from the search. Overall, the final literature search included 117 articles. After abstract screening, 94 articles were excluded. The full text of the remaining 24 articles and additional 12 articles which were identified through bibliography search were reviewed according to the inclusion criteria, and 13 articles were excluded. The search resulted in 22 articles being included in the meta-analysis (Fig. 1). Two authors (OO and AB) determined the final eligibility of studies, and any disagreements were resolved by the senior author (AB).

### Quality Assessment

The Newcastle–Ottawa Scale was used to assess the quality of the studies based on three aspects which are selection, comparability, and outcome or exposure. These three aspects are further divided into eight specific items which are representativeness of the exposed cohort, selection of the non-exposed cohort, ascertainment of exposure, demonstration that outcome of interest was not present at start of the study, comparability of cohorts on the basis of design, assessment method of outcome, whether follow-up was long enough for outcomes to occur, and the adequacy of follow-up of cohorts. Each item on the scale could get a score of one point except comparability which could get a score up to two points. Thus, overall scores in this scale could range from 0 to 9, with higher scores indicating higher quality. The scores of included studies ranged from 4 to 7 indicating good quality (Supplemental Table 1).

**Table 1:** Summary of the studies included in the analysis

First Author (Year)	N	% of Males	Maternal age (A: < 29; B: > = 30)	Country income level (High; Upper-Middle)	Prenatal depression assessment Point	Postnatal depression assessment point (A: < = 6 months; B: > 6 months)	Child Age (A: < 12 months; B: > = 12 months)	Name of depression measure	Sleep problems measure (A: validated questionnaire/Objective measure; B: One-item)
Alvik et al. [2]	1749	N/A	30.8 (B)	Norway (High)	30 weeks	N/A	6 months (A)	H-SCL	DTS and ITSC (A)
Bilgin and Wolke [11]	105	57%	30.7 (B)	UK (High)	N/A	6 months (A)	18 months (B)	EPDS	ISHQ & ISQ (A)
Chuang et al. [17]	186	57%	32.6 (B)	China (Upper Middle)	N/A	Birth (A)	24 months (B)	MHI-5	CBCL (A)
Dennis and Ross [20]	505	N/A	29 (A)	Canada (High)	N/A	4 weeks (A)	8 weeks (A)	EPDS	Questionnaire (8-items) devised for the study (A)
Dias and Figueiredo [21]	312	54%	N/A	Portugal (High)	3rd trimester	3 months (A)	6 months (A)	EPDS	CSHQ-I (A)
Galbally et al. [26]	264	55%	31.2 (B)	Australia (High)	3rd trimester	6 months (A)	12 months (B)	EPDS	BISQ (A)
Garthus-Niegel et al. [28]	1480	52%	31.7(B)	Norway (High)	32 weeks	8 weeks (A)	2 years (B)	EPDS	BISQ (A)
Goldberg et al. [30]	171	55%	28.9 (A)	America (High)	N/A	6 months (A)	12 months (B)	CES-D	SPQ (A)
Gress-Smith et al. [32]	132	N/A	26.5 (A)	America (High)	N/A	5 months (A)	9 months (A)	CES-D	1 item: ‘*how often baby woke during the night*’ (B)
Gui et al. [33]	243	51%	29.5 (A)	China (Upper Middle)	3rd trimester	42 days (A)	6 months (A)	CES-D (prenatal), EPDS (42 days), POMS (36 months)	BISQ (A)
Halal et al. [34]*	2222	50%	46.3% were 20–30 years (A)	Brazil (Upper Middle)	16–24 weeks	3 months (A)	12 months (A)	EPDS	BISQ & Actigraphy (A)
Kim (2020)	5568	N/A	N/A	New Zealand (High)	3rd trimester	9 months (B)	2 years (B)	EPDS	IBQ-R VSF (A)
Ma (2022)**	1583	53%	N/A	China (Upper Middle)	3rd trimester	3 months (A)	30 months (B)	CES-D & EPDS	BISQ (A)
Martini et al. [46]*	286	51%	28.1 (A)	Germany (High)	10 – 12 weeks	4 months (A)	16 months (B)	Computer-assisted personal interview version of the CIDI-V	Baby-DIPS (A)
Matenchuk et al. [47]	619	50%	N/A	Canada (High)	36 weeks	N/A	3 months (A)	CES-D	BISQ (A)
O’Connor et al. [51]	11,490	51%	28.5 (A)	UK (High)	32 weeks	8 months (B)	18 months (B)	EPDS	Sleep-index devised for the study (6 items) (A)
Pinheiro et al. [58]	397	N/A	26.2 (A)	Brazil (Upper Middle)	N/A	9–12 weeks (A)	12 months (B)	EPDS	1-item: ‘*sleep more than 12 h per day or less than 12 h per day, naps included*’ (B)
Simard et al. [65]	106	N/A	30.5 (B)	Canada (High)	N/A	6 weeks (A)	12 weeks (A)	EPDS	1-item: ‘How many hours does he/she sleep consecutively during the night?’ (B)
Simcock et al. [66]	134	54%	N/A	Australia (High)	Exact timing unclear	N/A	30 months (B)	EPDS and DASS-21	CBCL (A)
Tikotzky et al. [69]	226	51%	28.8 (A)	Israel (High)	3rd trimester	12 months (B)	18 months (B)	EPDS	BISQ (A)
Warren et al. [72]	1222	51%	28 (A)	America (High)	N/A	6 months (A)	15 months (B)	CES-D	1-item: ‘*In the last week has baby wakened you at night?”* (B)
Ystrom et al. [73]	14, 926	52%	28.6 (A)	Norway (High)	N/A	6 months (A)	18 months (B)	SCL-8	1-item: “How often does your child wake up nowadays?” (B)

Maternal age: we used the majority category when mean values were not provided

*Halal [34] and Martini et al. [46] reported on perinatal depression

**Only included in prenatal depression analysis given the larger sample size

*H-SCL* hopkins symptom checklist, *DTS* the difficult temperament scale of the infant characteristics questionnaire, *ITSC* infant toddler symptom checklist, *EPDS* Edinburgh postnatal depression scale, *ISHQ* infant sleep habits questionnaire, *ISQ* infant sleep Questionnaire, *MHI-5* mental health index, *CBCL/1.5–5* child behaviour checklist for ages 1.5–5, *CSHQ-I* children’s sleep habits questionnaire-infant version, *BISQ* brief infant sleep questionnaire, *CES-D* center for epidemiological studies depression inventory, *SPQ* sleep practices questionnaire, *POMS* profile of mood states, *IBQ-R VSF* very short form of infant behaviour questionnaire-revised, *CIDI-V* composite international diagnostic interview for women, *Baby-DIPS* diagnostic interview for regulatory problems, *DSM-IIIR* diagnostic and statistical manual of mental disorders 3rd edition revised, *LIFE* longitudinal interval follow-up evaluation, *HRSD* hamilton rating scale for depression, *CHQ-12* Chinese health questionnaire, *SHQ* sleep habit questionnaire, *DASS-21* depression, anxiety and stress scale, *SCL-8* hopkins symptom checklist

### Data Extraction

Eligible studies were reviewed to extract the relevant maternal and infant data, different studies provided the data in different formats: sample size with means and SDs, correlations, odds ratios. Categorical information regarding child age at assessment of sleep problems (up to 12 months of age or 12 months of age and above based on the mean age of the children in the study), maternal age (under 30 years or 30 years and above based on the mean age of the mothers in the study), income level of the country (high or upper middle income), assessment of sleep problems in children (valid questionnaire/objective measurement or one question), and time of assessment of postnatal depression (during the first 6 months or after 6 months) was extracted from the articles (Table 1). The data extraction was completed by OO and double-checked by AB, the senior author.

We used the following protocol so that each sample of participants was represented only once in the meta-analysis: 1) If more than one-time point of maternal depressive symptoms or child sleep problems were provided, effect sizes from the first time point will be used. This deviated from the preregistered data extraction protocol where we originally planned to pool the effect sizes across different time points if more than one-time point of assessments were made. The reason for this change was to extract the effect size from the largest sample size. 2) If multiple publications emerged from a dataset, we selected the publication with the largest sample size and most comprehensive data extraction information.

### Data Analysis

Analyses to test for overall effect sizes, publication bias, and potential moderators were conducted using Comprehensive Meta Analysis software (version 4). Effect sizes of individual studies were transformed into odds ratios (OR), and 95% CI. Effect size calculations were based on random effects modeling which uses the assumption that each study has its own population parameters. Thus, random-effects models take into account that effect sizes will differ from one study to another because they are sampled from an unknown distribution [13]. Heterogeneity of studies was assessed with Cochran’s Q and Higgins *I*^2^. A significant Q statistic suggests that study variability in effect size estimates is greater than sampling error and that moderators should be explored. The *I*^2^ statistic examines the rate of variability across studies due to heterogeneity rather than chance, with values of 50% and 75% or above suggesting moderate to high heterogeneity respectively [35]. To examine whether moderators could explain variability across studies, categorical moderator analyses were conducted. Moderator analyses included dividing the studies into subgroups based on the following variables: child age at assessment of sleep problems (up to 12 months of age or 12 months of age and above), maternal age (under 30 years or 30 years and above), income level of the country (high or upper middle income), assessment of sleep problems in children (valid questionnaire/objective measurement or one question), and assessment time of maternal depression in the postnatal period (during the first 6 months, after 6 months). Different subgroups might contain different number of data points (i.e., number of studies included in a subgroup), and thus might have different abilities to detect significant effects. If one subgroup analysis reveals significant findings but the other subgroup does not, this might simply reflect a lack of enough data points (i.e., number of studies included in a subgroup) rather than smaller or absent effect. Thus, it may be misleading to simply compare the p-values and magnitude of the effect sizes across subgroups. To test whether any differences between subgroups exist, a standard test for heterogeneity across subgroups using Cochran’s Q was undertaken for all moderators.

Publication bias analysis was assessed by using 3 strategies. First, the trim and fill procedure [23] was used to examine the symmetry of effect sizes plotted by the inverse of the SE. Ideally, the effect sizes should mirror one another on either side of the mean. Second, the Begg and Mazumdar rank correlation test [8] was used to examine the likelihood of bias in favor of small sample size studies. Nonsignificance of correlation indicates no publication bias. Last, Egger’s test [24] examined whether publication bias related to the direction of study findings. The intercept value provided by this test shows the level of funnel plot asymmetry from the standard precision.

Further, we ran several sensitivity analyses. First, we repeated the main analysis investigating the association between prenatal depression and childhood sleep problems excluding Simcock et al. [66] as the assessment point of depression during pregnancy was not clear and was extracted from hospital records. Second, we repeated the main analysis investigating the association between prenatal depression and childhood sleep problems excluding Halal et al. [34] and Martini et al. [46] as these studies reported on perinatal depression (combined depression score during and after pregnancy). Third, we repeated the meta-analysis on the association between postnatal depression and child sleep problems excluding the studies that did not include assessments of depression during the prenatal period [21, 26, 33, 41, 51, 69] to check whether controlling for the effects of prenatal depression altered the strength of the association between postnatal depression and sleep problems in early childhood.

## Results

The 22 studies of maternal depression and sleep problems in early childhood represented a total of 43,926 participants. Twelve studies contained reports of prenatal depression and 16 of them contained a report of postnatal depression, while 6 of them contained a report of both prenatal and postnatal depression. The age of children at assessment ranged from 8 weeks to 30 months. Eight studies included assessments of infants aged 12 months and below, while 14 studies assessed infants who are 12 months or older. The age of mothers ranged from 26.2 to 32.6 years with 12 studies including mothers aged 29 years or younger and 6 including mothers older than 30 years. The information on maternal age was not provided for the remaining 5 studies. Most of the studies were conducted in high income countries (N = 17), whilst the remaining studies were conducted in upper-middle income countries. Most studies used valid measurement of sleep problems (N = 17) and the majority of the studies assessed postnatal depression during the first 6 months (N = 13).

### Associations Between Prenatal Depression and Sleep Problems in Early Childhood

**Fig. 2:**
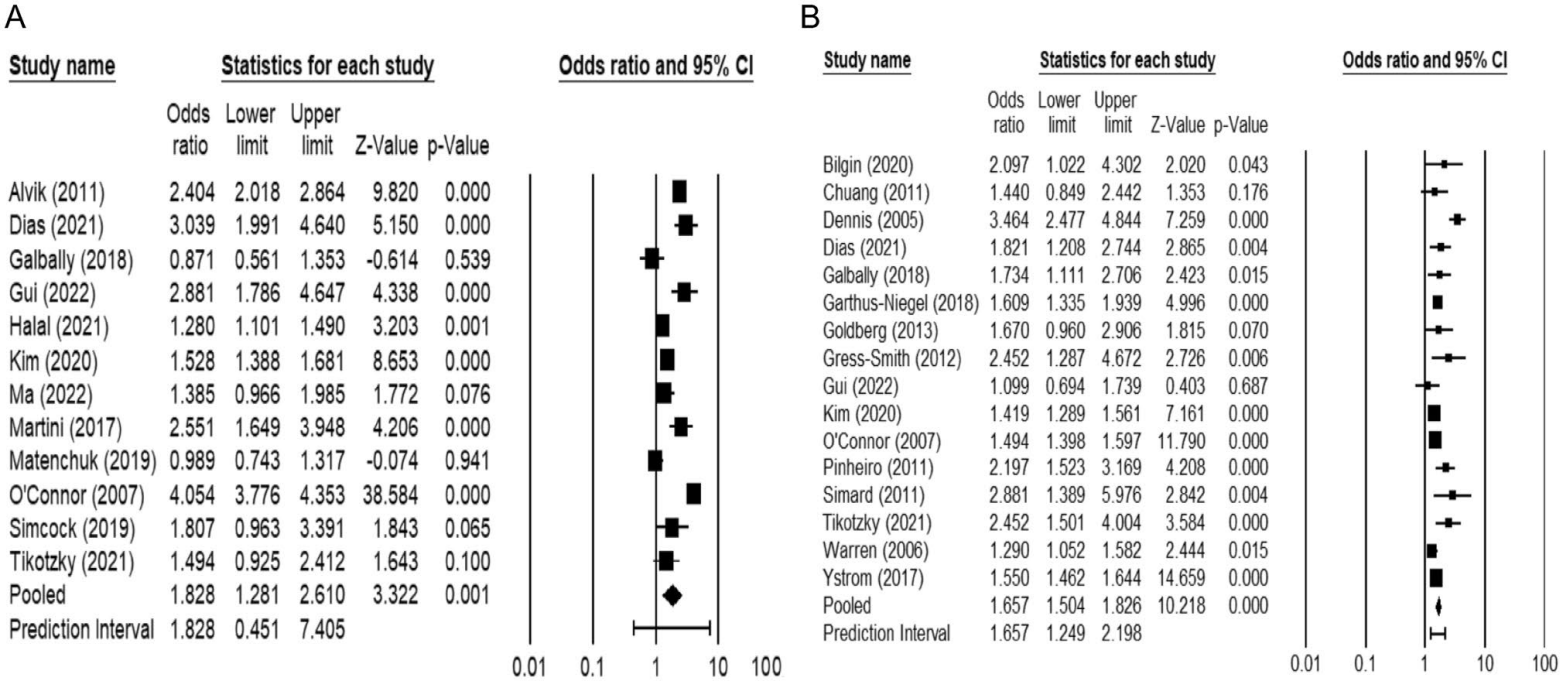
Forest plots of the associations between **A** prenatal depression, **B** postnatal depression and sleep problems in early childhood

There was a significant positive association between prenatal depression and sleep problems in early childhood (OR = 1.828; 95% CI = 1.281–2.610), indicating that prenatal depression was associated with an increase in the likelihood of sleep problems in early childhood (Table 2; Fig. 2). Heterogeneity analysis indicated significant and high variation between studies (*Q* = 432.323; *I*^2^ = 97.456, *P* < 0.001). Subgroup analysis according to the child age at assessment of sleep problems did not reveal any between group differences (*Q* = 0.344,* p* = 0.557). There was a significant association between prenatal depression and sleep problems both for children aged below 12 months (OR = 2.102; 95% CI = 1.250–3.535), and for children who were aged 12 months and above (OR = 1.698; 95% CI = 1.059–2.723). Similarly, subgroup analysis based on maternal age did not reveal any between group differences (*Q* = 0.617, *p* = 0.432). However, there was a significant association between prenatal depression and sleep problems in early childhood for the mothers were aged below 30 years (OR = 2.075; 95% CI = 1.149–3.746), but not for mothers who were 30 years or older (OR = 1.592; 95% CI = 0.889- 2.849). Subgroup analysis based on country income level did not reveal any between group differences (*Q* = 0.036, *p* = 0.848). There was a significant association between prenatal depression and sleep problems in early childhood similarly in high income (OR = 1.876; 95% CI = 1.237–2.847) and upper-middle income countries (OR = 1.638; 95% CI = 1.083–2.476).

**Table 2:** Associations between maternal depression and sleep problems in early childhood

	Data points	OR	95% CI Lower bound	95% CI Upper bound	Cochran Q test	*I*^2^	Test for heterogeneity (*P*)
Prenatal depression and sleep problems in early childhood							
All studies	12	1.828	1.281	2.610	432.323	97.456	< 0.001
Child age					0.344		0.557
Below 12 months	4	2.102	1.250	3.535	33.310	90.994	< 0.001
12 months and above	8	1.698	1.059	2.723	391.798	98.213	< 0.001
Maternal age					0.617		0.432
Below 30 years	6	2.075	1.149	3.746	214.732	97.672	< 0.001
30 years and above	4	1.592	0.889	2.849	43.466	93.098	< 0.001
Country income level					0.036		0.848
High	9	1.876	1.237	2.847	342.508	97.664	< 0.001
Upper middle	3	1.638	1.083	2.476	10.044	80.088	0.007
Postnatal depression and sleep problems in early childhood							
All studies	16	1.657	1.504	1.826	44.902	65.594	< 0.001
Child age					2.264		0.132
Below 12 months	5	2.156	1.375	3.381	17.242	76.801	0.002
12 months and above	11	1.516	1.460	1.574	14.288	30.013	0.160
Maternal age					0.024		0.877
Below 30 years	9	1.718	1.491	1.979	37.346	78.579	< 0.001
30 years and above	6	1.690	1.461	1.955	3.157	0.000	0.676
Country income level					0.100		0.752
High	13	1.661	1.502	1.838	39.136	69.338	< 0.001
Upper middle	3	1.547	1.004	2.382	5.588	64.206	0.061
Sleep problems assessment					0.000		0.996
Valid measure	11	1.695	1.475	1.947	33.331	69.998	< 0.001
One-item	5	1.696	1.376	2.092	11.298	64.595	0.023
Postnatal depression assessment time					3.013		0.083
During the first 6 months	13	1.766	1.517	2.057	36.512	67.134	< 0.001
After 6 months	3	1.495	1.339	1.669	4.890	59.098	0.087

Please note that sleep problems assessment subgroup analysis was not conducted for prenatal depression since all measurements were made with valid questionnaires

### Associations Between Postnatal Depression and Sleep Problems in Early Childhood

There was a significant positive association between postnatal depression and sleep problems in early childhood (OR = 1.657; 95% CI = 1.504–1.826), suggesting that postnatal depression was associated with an increase in the likelihood of sleep problems in early childhood (Table 2; Fig. 2). Heterogeneity analysis indicated significant and high variation between studies (*Q* = 44.902, *I*^2^ = 65.594, *P* < 0.001). Subgroup analysis according to child age at assessment of sleep problems did not reveal any between group differences (*Q* = 2.264, *p* = 0.132). There was an association between postnatal depression and sleep problems both for children who are aged below 12 months (OR = 2.156; 95% CI = 1.375–3.381) and 12 months or older (OR = 1.516; 95% CI = 1.460–1.574). Similarly, subgroup analysis based on maternal age did not reveal any between group differences (*Q* = 0.024, *p* = 0.877). There was a significant association between postnatal depression and sleep problems in children for both mothers who were younger than 30 years (OR = 1.718; 95% CI = 1.491–1.979) and those who were aged 30 and above (OR = 1.690; 95% CI = 1.461–1.955). Further, subgroup analysis on country income did not reveal any between group differences (*Q* = 0.100, *p* = 0.752). Studies from both high (OR = 1.661; 95% CI = 1.502–1.838) and upper-middle income countries (OR = 1.547; 95% CI = 1.004–2.382) revealed an association between postnatal depression and sleep problems in early childhood. Moreover, subgroup analysis on the measurement of sleep problems did not reveal any between group differences (*Q* = 0.000, *p* = 0.996). Studies using both valid measurements (OR = 1.695; 95% CI = 1.475–1.947) and one-item questions (OR = 1.696; 95% CI = 1.376–2.092) revealed an association between postnatal depression and sleep problems in early childhood. Finally, subgroup analysis based on the time of assessment of maternal depression did not reveal any between group differences (*Q* = 3.013, *p* = 0.083). There was a significant association between postnatal depression and sleep problems in children both when depression was assessed during the first 6 months (OR = 1.766; 95% CI = 1.517–2.057), and after 6 months (OR = 1.495; 95% CI = 1.339–1.669).

### Publication *Bias*

Regarding the association between prenatal depression and sleep problems in early childhood, the point estimate (95% CI) for the combined studies is 1.828 (1.280–2.609) under the random effects model. With the use of trim and fill, these values remained unchanged, indicating no publication bias. The Begg and Mazumdar rank correlation and Egger et al.’s test were not statistically significant, indicating no evidence for publication bias.

Regarding the association between postnatal depression and sleep problems in early childhood, the point estimate (95% CI) for the combined studies is 1.657 (1.504–1.826) under the random effects model. Using trim and fill, the imputed point estimate is 1.504 (1.349–1.678), which is the point estimate adjusted for publication bias. This suggests that our initial findings regarding the association between postnatal depression and sleep problems in early childhood may be slightly overestimated due to publication bias. However, it is important to note that there is no guarantee that the adjusted point estimate equals to what would have been observed in the absence of publication bias given that we cannot know the true mechanism behind the publication bias. Further, it has been suggested that the trim and fill method performs poorly when there is high heterogeneity between studies [57]. Thus, the corrected point estimate should be interpreted with caution. The Begg and Mazumdar rank correlation test and Egger et al.’s test were not statistically significant, indicating no publication bias.

### Sensitivity Analysis

We repeated the main analyses for the association between prenatal depression and sleep problems excluding Simcock et al. [66] which revealed a similar estimate (OR = 1.830,95% CI: 1.262–2.652). Second, we repeated the main analysis investigating the association between prenatal depression and childhood sleep problems excluding Halal et al. [34] and Martini et al. [46] as these studies reported on perinatal depression (combined depression score during and after pregnancy) which revealed a similar estimate (OR = 1.839,95% CI: 1.239–2.730).

When we repeated the meta-analysis on the association between postnatal depression and child sleep problems excluding the studies that did not include assessments of depression during the prenatal period (i.e., only including studies which adjusted for the role of prenatal depression), there was still a significant association between postnatal depression and child sleep problems although the strength of the association was smaller (OR = 1.499; 95% CI = 1.359; 1.654).

## Discussion

### Main Findings

Findings of the current meta-analysis showed that maternal depressive symptoms during both prenatal and postnatal periods are associated with increased likelihood of having a child with sleep problems. Depressive symptoms during pregnancy were associated with 1.82 increased likelihood of sleep problems in children, while depressive symptoms during the postnatal period increased the likelihood of sleep problems in early childhood by 1.65. When we repeated the meta-analysis on the association between postnatal depression and child sleep problems only focusing on the studies which adjusted for the role of prenatal depression, the likelihood of having a child with sleep problems was 1.49. These associations for both prenatal and postnatal depression were consistent across child and maternal age at assessment, income level of the countries, and the assessment type of sleep problems in childhood.

Current findings highlight that there is an 82% increase in the odds of developing childhood sleep problems when mothers show depressive symptoms during pregnancy [21, 51]. This association was consistent across child and maternal age at assessment, and income level of the countries. The mechanism underlying this association could be explained via the Developmental Origins of Health and Disease (DOHaD) hypothesis, which posits that adverse environmental events during the pregnancy period leads to significant consequences on long term health including psychopathology [6]. In line with this hypothesis, during pregnancy, the fetus could receive signals of stress caused by depression which could have persisting influences on the brain development and physiological development [59]. One physiological mechanism is dysregulation in the development of the hypothalamic–pituitary–adrenal (HPA) axis, which is linked to the development of systems responsible for regulating circadian rhythms [75]. Thus, in the context of infant sleep problems early adverse effects can lead to an overactive HPA axis, which can distort circadian sleep–wake cycles and lead to increased infant wakefulness [53, 70].

Our findings showed that there is a 65% increase in the odds of developing childhood sleep problems when mothers show depressive symptoms during the postnatal period, which is consistent across assessment age in childhood (below 12 months or 12 months and above), maternal age (below 30 years or 30 years and above), country income level (high or upper-middle), the type of measurement used to assess of sleep (valid questionnaire or one-item question), and time of assessment of postnatal depression (before 6 months of age or after 6 months). When the analysis was repeated only focusing on the studies which adjusted for the role of prenatal depression, maternal depressive symptoms during the postnatal period increased the likelihood of sleep problems in early childhood by 49%. The decrease in the likelihood of sleep problems in early childhood from 65 to 49% could be due to the impact of the correlation between prenatal and postnatal depression symptoms given that prenatal depression symptoms is a key risk factor for postnatal depression symptoms [48].

It was suggested that the link between maternal depression and sleep problems in children could be bidirectional in the postnatal period [38]. Regarding the impact of maternal depression on the sleep problems of their offspring, there is evidence that symptoms of maternal depression are associated with having difficulties in setting a bedtime routine and being in tune with babies’ signals which could contribute to sleep problems in early childhood [36]. Further, it is possible that mothers with depressive symptoms have a higher tendency to report and be concerned by sleep problems of their children than mothers who do not show depressive symptoms. On the other hand, child sleep problems could impact maternal postnatal depression symptoms through increasing sleep deprivation in mothers. In line with this argument, intervention studies showed that implementing nighttime parenting strategies to reduce infant sleep difficulties also reduces parents’ depressive symptoms [37]. However, the existing evidence from prospective longitudinal studies which investigated the bidirectional association between maternal depression and child sleep problems so far suggests that maternal depression symptoms during the postnatal period influences child sleep problems rather than the reverse association [21].

Given the adverse long-term impact of childhood sleep problems, it is important to identify those most at risk. The current meta-analysis suggests a large influence of maternal depressive symptoms during the prenatal period and a moderate influence of depression during the postnatal period on the development of sleep problems in children. However, it is important to highlight the complexity of how sleep problems develop in children. The theoretical model proposed by Sadeh and Anders [60] suggests that the development of sleep problems is influenced by factors at the distal extrinsic context (e.g., social and cultural norms, caretaking arrangements, family stress), proximal extrinsic context (e.g., parental personality), intrinsic context (e.g., infant temperament), as well as parent-infant mediating context (e.g., bedtime interaction, soothing behaviors, bedsharing). Thus, maternal depressive symptoms are likely to interact with other factors, which were not explored in the current meta-analysis. Further, sleep problems are also a symptom of depression which adds to the complexity of understanding the association between maternal depression and child sleep problems within and across generations [4].

It is also possible that the association between maternal depressive symptoms and sleep problems in children is due to unmeasured genetic confounding. To illustrate, findings from recent meta-analyses focusing on twin studies suggested substantial heritability rates for insomnia is 40% [5]. It was shown that the genetic predisposition for insomnia in adulthood is related to early childhood (at 1.5 years) sleep problems (i.e., frequent awakening, difficulty initiating sleep) reported by the mother [42]. Thus, future studies could focus on genetically sensitive designs adjusting for polygenetic risk scores or using sibling comparisons of differentially exposed siblings to elucidate the role of genetics on this association.

Prior to discussion of the clinical implications of our findings, it is important to note that the findings of the meta-analyses are correlational in nature. Causality between maternal depression and sleep problems in early childhood cannot be established based on our findings. However, there is increasing awareness of the importance of early intervention services during pregnancy to mitigate the depression symptoms of mothers to promote healthy fetal development. It is likely that investing in interventions to decrease maternal depression symptoms will have broad-reaching benefits on the health of mothers, their children and their relationship.

### Limitations 

There are some limitations of the current meta-analysis. First, we only included articles which were published in English. We cannot be certain whether this introduced bias, however, no significant publication bias was found for the studies in the current analysis. Second, there were too few studies from upper-middle income and no studies from low-income countries. These would be needed to understand the impact of maternal depression on early childhood sleep problems in all regions of the world. Third, the heterogeneity was high which indicates variation between the included studies. This heterogeneity might be related to inclusion of studies that have different methodology. To address this, we used random-effects model in the analysis and conducted moderator analysis with potential variables. Nevertheless, our moderator analysis explained only some of the heterogeneity. Thus, the findings from the current study should be interpreted with caution and the analyses should be repeated when more data becomes available from other cohort studies. Fourth, the majority of the studies included in the current analysis used maternal reports rather than objective measurements such as actigraphy, which may be due to difficulties of implementing objective measurements in longitudinal prospective studies. Thus, the current analysis should be repeated in the future when more data on objective sleep measurements is available to compare the findings of studies using parental questionnaires to objective measurements.

Future studies could explore the role of the timing of prenatal depression assessment on the association between maternal depression and sleep problems in early childhood. We were unable to explore the role of the timing of prenatal depression assessment in the current analysis since all prenatal studies included in this review screened for depression during the third trimester as per clinical recommendations. Moreover, future studies could explore if the association between maternal depressive symptoms and sleep problems in early childhood differs according to children’s sex. Further, parental practices and expectations regarding child sleep and what constitutes a sleep problem could vary depending on cultural values [40]. To illustrate, co-sleeping (i.e., bed- or room- sharing) is more common in non-western societies, low-income, and minority families [18]. In particular, mothers who bed-share may be more predisposed to show higher depressive symptoms than those who do not bed-share [50]. Further, there is evidence that bed-sharing predicts increased sleep problems in infants [11]. Thus, co-sleeping could moderate the association between maternal postnatal depression and sleep problems in early childhood.

## Summary

Our findings show that the strength of the association between maternal depression and sleep problems in early childhood is very large, with 82% increased likelihood of sleep problems in children whose mothers had prenatal depression, and 65% increased likelihood of sleep problems in children whose mothers had postnatal depression. There was high heterogeneity between the studies which mean that conclusions are tentative and need to be considered within the possible influence of unmeasured confounding. However, mitigating depression symptoms in mothers both during pregnancy and in the postnatal period would be an effective strategy for reducing sleep problems in children.

## Data Availability

Data sharing is not applicable to this article as no datasets were generated.

## References

[1] Agnafors S, Bladh M, Svedin CG, Sydsjö G (2019) Mental health in young mothers, single mothers and their children. BMC Psychiatry 19(1):112. 10.1186/s12888-019-2082-y 30975129 10.1186/s12888-019-2082-y PMC6460673

[2] Alvik A, Torgersen AM, Aalen OO, Lindemann R (2011) Binge alcohol exposure once a week in early pregnancy predicts temperament and sleeping problems in the infant. Early Human Dev 87(12):827–833. 10.1016/j.earlhumdev.2011.06.009 10.1016/j.earlhumdev.2011.06.009 21757302

[3] Armitage R, Flynn H, Hoffmann R, Vazquez D, Lopez J, Marcus S (2009) Early developmental changes in sleep in infants: the impact of maternal depression. Sleep 32(5):693–696. 10.1093/sleep/32.5.693 19480236 10.1093/sleep/32.5.693 PMC2675904

[4] Baglioni C, Battagliese G, Feige B, Spiegelhalder K, Nissen C, Voderholzer U (2011) Insomnia as a predictor of depression: a meta-analytic evaluation of longitudinal epidemiological studies. J Affect Disord 135:10–19. 10.1016/j.jad.2011.01.011 21300408 10.1016/j.jad.2011.01.011

[5] Barclay NL, Kocevska D, Bramer WM, Van Someren EJW, Gehrman P (2021) The heritability of insomnia: a meta-analysis of twin studies. Genes Brain Behav 20(4):e12717. 10.1111/gbb.12717 33222383 10.1111/gbb.12717

[6] Barker DJ (2007) The origins of the developmental origins theory. J Intern Med 261(5):412–417. 10.1111/j.1365-2796.2007.01809.x 17444880 10.1111/j.1365-2796.2007.01809.x

[7] Bayer JK, Hiscock H, Hampton A, Wake M (2007) Sleep problems in young infants and maternal mental and physical health. J Paediatr Child Health 43(1–2):66–73. 10.1111/j.1440-1754.2007.01005.x 17207059 10.1111/j.1440-1754.2007.01005.x

[8] Begg CB, Mazumdar M (1994) Operating characteristics of a rank correlation test for publication bias. Biometrics 50(4):1088–1101 7786990

[9] Bernier A, Beauchamp MH, Bouvette-Turcot A-A, Carlson SM, Carrier J (2013) Sleep and Cognition in Preschool Years: Specific Links to Executive Functioning. Child Dev 84(5):1542–1553. 10.1111/cdev.12063 23432661 10.1111/cdev.12063

[10] Bilgin A, Wolke D (2020) Infant crying problems and symptoms of sleeping problems predict attachment disorganization at 18 months. Attach Hum Dev 22(4):367–391. 10.1080/14616734.2019.1618882 31132936 10.1080/14616734.2019.1618882

[11] Bilgin A, Wolke D (2022) Bed-sharing in the first 6 months: associations with infant-mother attachment, infant attention, maternal bonding, and sensitivity at 18 months. J Dev Behav Pediatr 43(1):e9–e19. 10.1097/dbp.0000000000000966 34117203 10.1097/DBP.0000000000000966

[12] Black MM, Walker SP, Fernald LCH, Andersen CT, DiGirolamo AM, Lu C, McCoy DC, Fink G, Shawar YR, Shiffman J, Devercelli AE, Wodon QT, Vargas-Barón E, Grantham-McGregor S (2017) Early childhood development coming of age: science through the life course. The Lancet 389(10064):77–90. 10.1016/S0140-6736(16)31389-7 10.1016/S0140-6736(16)31389-7 PMC5884058 27717614

[13] Borenstein M, Hedges LV, Higgins JPT, Rothstein HR (2009) Introduction to meta- analysis. John Wiley & Sons

[14] Burdayron R, Butler BP, Béliveau MJ, Dubois-Comtois K, Pennestri MH (2021) Perception of infant sleep problems: the role of negative affectivity and maternal depression. J Clin Sleep Med 17(6):1279–1285. 10.5664/jcsm.9188 33660614 10.5664/jcsm.9188 PMC8314649

[15] Byars KC, Yolton K, Rausch J, Lanphear B, Beebe DW (2012) Prevalence, patterns, and persistence of sleep problems in the first 3 years of life. Pediatrics 129(2):e276-284. 10.1542/peds.2011-0372 22218837 10.1542/peds.2011-0372 PMC3357046

[16] Christian LM, Franco A, Glaser R, Iams JD (2009) Depressive symptoms are associated with elevated serum proinflammatory cytokines among pregnant women. Brain Behav Immun 23(6):750–754. 10.1016/j.bbi.2009.02.012 19258033 10.1016/j.bbi.2009.02.012 PMC2710424

[17] Chuang CH, Jeng SF, Hsieh WS, Liao HF, Su YN, Chen PC (2011) Maternal psychosocial factors around delivery, and the behavior of 2-year-old children. Pediatr Int 53(5):656–661. 10.1111/j.1442-200X.2010.03315.x 21199165 10.1111/j.1442-200X.2010.03315.x

[18] Colson ER, Willinger M, Rybin D, Heeren T, Smith LA, Lister G, Corwin MJ (2013) Trends and factors associated with infant bed sharing, 1993–2010: the national infant sleep position study. JAMA Pediatr 167(11):1032–1037. 10.1001/jamapediatrics.2013.2560 24080961 10.1001/jamapediatrics.2013.2560 PMC3903787

[19] Dahl RE (2007) Sleep and the developing brain. Sleep 30(9):1079–1080. 10.1093/sleep/30.9.1079 17910377 10.1093/sleep/30.9.1079 PMC1978403

[20] Dennis CL, Ross L (2005) Relationships among infant sleep patterns, maternal fatigue, and development of depressive symptomatology. Birth 32(3):187–193. 10.1111/j.0730-7659.2005.00368.x 16128972 10.1111/j.0730-7659.2005.00368.x

[21] Dias CC, Figueiredo B (2021) Unidirectional and bidirectional links between maternal depression symptoms and infant sleep problems. J Sleep Res 30(5):e13363. 10.1111/jsr.13363 33900005 10.1111/jsr.13363

[22] Dionne G, Touchette E, Forget-Dubois N, Petit D, Tremblay RE, Montplaisir JY, Boivin M (2011) Associations between sleep-wake consolidation and language development in early childhood: a longitudinal twin study. Sleep 34(8):987–995. 10.5665/sleep.1148 21804661 10.5665/SLEEP.1148 PMC3138173

[23] Duval S, Tweedie R (2000) A nonparametric, “trim and fill” method of accounting for publication bias in meta-analysis. J Am Stat Assoc 95(449):89–98. 10.1080/01621459.2000.10473905

[24] Egger M, Davey Smith G, Schneider M, Minder C (1997) Bias in meta-analysis detected by a simple, graphical test. BMJ 315(7109):629–634. 10.1136/bmj.315.7109.629 9310563 10.1136/bmj.315.7109.629 PMC2127453

[25] Fergusson DM, Woodward LJ (1999) Maternal age and educational and psychosocial outcomes in early adulthood. J Child Psychol Psychiatry 40(3):479–489 10190348

[26] Galbally M, Watson SJ, Teti D, Lewis AJ (2018) Perinatal maternal depression, antidepressant use and infant sleep outcomes: exploring cross-lagged associations in a pregnancy cohort study. J Affect Disord 238:218–225. 10.1016/j.jad.2018.05.025 29886202 10.1016/j.jad.2018.05.025

[27] Galland BC, Taylor BJ, Elder DE, Herbison P (2012) Normal sleep patterns in infants and children: a systematic review of observational studies. Sleep Med Rev 16(3):213–222. 10.1016/j.smrv.2011.06.001 21784676 10.1016/j.smrv.2011.06.001

[28] Garthus-Niegel S, Horsch A, Bickle Graz M, Martini J, von Soest T, Weidner K, Eberhard-Gran M (2018) The prospective relationship between postpartum PTSD and child sleep: A 2-year follow-up study. J Affect Disord 241:71–79. 10.1016/j.jad.2018.07.067 30098473 10.1016/j.jad.2018.07.067

[29] Glover V (2011) Annual research review: prenatal stress and the origins of psychopathology: an evolutionary perspective. J Child Psychol Psychiatry 52(4):356–367. 10.1111/j.1469-7610.2011.02371.x 21250994 10.1111/j.1469-7610.2011.02371.x

[30] Goldberg WA, Lucas-Thompson RG, Germo GR, Keller MA, Davis EP, Sandman CA (2013) Eye of the beholder? maternal mental health and the quality of infant sleep. Soc Sci Med 79:101–108. 10.1016/j.socscimed.2012.07.006 22858167 10.1016/j.socscimed.2012.07.006 PMC3540198

[31] Goodman SH, Gotlib IH (1999) Risk for psychopathology in the children of depressed mothers: a developmental model for understanding mechanisms of transmission. Psychol Rev 106(3):458–490. 10.1037/0033-295x.106.3.458 10467895 10.1037/0033-295x.106.3.458

[32] Gress-Smith JL, Luecken LJ, Lemery-Chalfant K, Howe R (2012) Postpartum depression prevalence and impact on infant health, weight, and sleep in low-income and ethnic minority women and infants. Matern Child Health J 16(4):887–893. 10.1007/s10995-011-0812-y 21559774 10.1007/s10995-011-0812-y

[33] Gui Y, Deng Y, Sun X, Li W, Rong T, Wang X, Jiang Y, Zhu Q, Liu J, Wang G, Jiang F (2022) Early childhood sleep trajectories and association with maternal depression: a prospective cohort study. Sleep. 10.1093/sleep/zsac037 35554573 10.1093/sleep/zsac037

[34] Halal CS, Bassani DG, Santos IS, Tovo-Rodrigues L, Del-Ponte B, Silveira MF, Bertoldi AD, Barros FC, Nunes ML (2021) Maternal perinatal depression and infant sleep problems at 1 year of age: subjective and actigraphy data from a population-based birth cohort study. J Sleep Res 30(2):e13047. 10.1111/jsr.13047 32285520 10.1111/jsr.13047

[35] Higgins JP, Thompson SG, Deeks JJ, Altman DG (2003) Measuring inconsistency in meta-analyses. BMJ 327(7414):557–560. 10.1136/bmj.327.7414.557 12958120 10.1136/bmj.327.7414.557 PMC192859

[36] Higley E, Dozier M (2009) Nighttime maternal responsiveness and infant attachment at one year. Attach Hum Dev 11(4):347–363. 10.1080/14616730903016979 19603300 10.1080/14616730903016979 PMC3422632

[37] Hiscock H, Bayer J, Gold L, Hampton A, Ukoumunne OC, Wake M (2007) Improving infant sleep and maternal mental health: a cluster randomised trial. Arch Dis Child 92(11):952–958. 10.1136/adc.2006.099812 17158146 10.1136/adc.2006.099812 PMC2083609

[38] Hiscock H, Wake M (2001) Infant sleep problems and postnatal depression: a community-based study. Pediatrics 107(6):1317–1322. 10.1542/peds.107.6.1317 11389250 10.1542/peds.107.6.1317

[39] Howard LM, Molyneaux E, Dennis C-L, Rochat T, Stein A, Milgrom J (2014) Non-psychotic mental disorders in the perinatal period. The Lancet 384(9956):1775–1788. 10.1016/S0140-6736(14)61276-9 10.1016/S0140-6736(14)61276-9 25455248

[40] Jenni OG, O’Connor BB (2005) Children’s sleep: an interplay between culture and biology. Pediatrics 115(1 Suppl):204–216. 10.1542/peds.2004-0815B 15866854 10.1542/peds.2004-0815B

[41] Kim Y, Bird A, Peterson E, Underwood L, Morton SMB, Grant CC (2020) Maternal antenatal depression and early childhood sleep: potential pathways through infant temperament. J Pediatr Psychol 45(2):203–217. 10.1093/jpepsy/jsaa001 32053187 10.1093/jpepsy/jsaa001

[42] Kocevska D, Trajanoska K, Mulder RH, Koopman-Verhoeff ME, Luik AI, Tiemeier H, van Someren EJW (2023) Are some children genetically predisposed to poor sleep? a polygenic risk study in the general population. J Child Psychol Psychiatry 65(5):710–719. 10.1111/jcpp.13899 37936537 10.1111/jcpp.13899

[43] Leis JA, Heron J, Stuart EA, Mendelson T (2014) Associations between maternal mental health and child emotional and behavioral problems: does prenatal mental health matter? J Abnorm Child Psychol 42(1):161–171. 10.1007/s10802-013-9766-4 23748337 10.1007/s10802-013-9766-4

[44] Lewandowski AS, Toliver-Sokol M, Palermo TM (2011) Evidence-based review of subjective pediatric sleep measures. J Pediatr Psychol 36(7):780–793. 10.1093/jpepsy/jsq119 21227912 10.1093/jpepsy/jsq119 PMC3146754

[45] Ma S, Yin X, Tao R, Jiang X, Xie J, Li P, Zhu D, Zhu P (2022) Association of maternal prenatal depression and anxiety with toddler sleep: the China-Anhui Birth Cohort study. Arch Womens Ment Health 25(2):431–439. 10.1007/s00737-021-01200-w 34997848 10.1007/s00737-021-01200-w

[46] Martini J, Petzoldt J, Knappe S, Garthus-Niegel S, Asselmann E, Wittchen HU (2017) Infant, maternal, and familial predictors and correlates of regulatory problems in early infancy: the differential role of infant temperament and maternal anxiety and depression. Early Hum Dev 115:23–31. 10.1016/j.earlhumdev.2017.08.005 28869923 10.1016/j.earlhumdev.2017.08.005

[47] Matenchuk BA, Tamana SK, Lou WYW, Lefebvre DL, Sears MR, Becker AB, Azad MB, Moraes TJ, Turvey SE, Subbarao P, Kozyrskyj AL, Mandhane PJ (2019) Prenatal depression and birth mode sequentially mediate maternal education’s influence on infant sleep duration. Sleep Med 59:24–32. 10.1016/j.sleep.2019.01.015 31153013 10.1016/j.sleep.2019.01.015

[48] Milgrom J, Gemmill AW, Bilszta JL, Hayes B, Barnett B, Brooks J, Ericksen J, Ellwood D, Buist A (2008) Antenatal risk factors for postnatal depression: a large prospective study. J Affect Disord 108(1–2):147–157. 10.1016/j.jad.2007.10.014 18067974 10.1016/j.jad.2007.10.014

[49] Moher D, Liberati A, Tetzlaff J, Altman DG (2009) Preferred reporting items for systematic reviews and meta-analyses: the PRISMA statement. BMJ 339:b2535. 10.1136/bmj.b2535 19622551 10.1136/bmj.b2535 PMC2714657

[50] Nulty AK, Thompson AL, Wasser HM, Bentley ME (2022) Directionality of the associations between bedsharing, maternal depressive symptoms, and infant sleep during the first 15 months of life. Sleep Health 8(1):39–46. 10.1016/j.sleh.2021.11.001 34922857 10.1016/j.sleh.2021.11.001 PMC8821130

[51] O’Connor TG, Caprariello P, Blackmore ER, Gregory AM, Glover V, Fleming P (2007) Prenatal mood disturbance predicts sleep problems in infancy and toddlerhood. Early Hum Dev 83(7):451–458. 10.1016/j.earlhumdev.2006.08.006 17008033 10.1016/j.earlhumdev.2006.08.006 PMC2853892

[52] Owens J (2008) Classification and epidemiology of childhood sleep disorders. Prim Care 35(3):533–546. 10.1016/j.pop.2008.06.003 18710669 10.1016/j.pop.2008.06.003

[53] Palagini L, Drake CL, Gehrman P, Meerlo P, Riemann D (2015) Early-life origin of adult insomnia: does prenatal–early-life stress play a role? Sleep Med 16(4):446–456 25799941 10.1016/j.sleep.2014.10.013

[54] Palma-Gudiel H, Córdova-Palomera A, Eixarch E, Deuschle M, Fañanás L (2015) Maternal psychosocial stress during pregnancy alters the epigenetic signature of the glucocorticoid receptor gene promoter in their offspring: a meta-analysis. Epigenetics 10(10):893–902. 10.1080/15592294.2015.1088630 26327302 10.1080/15592294.2015.1088630 PMC4844196

[55] Parsons CE, Young KS, Rochat TJ, Kringelbach ML, Stein A (2012) Postnatal depression and its effects on child development: a review of evidence from low- and middle-income countries. Br Med Bull 101:57–79. 10.1093/bmb/ldr047 22130907 10.1093/bmb/ldr047

[56] Pennestri M-H, Laganière C, Bouvette-Turcot A-A, Pokhvisneva I, Steiner M (2018) Uninterrupted Infant Sleep, Development, and Maternal Mood. Pediatrics. 10.1542/peds.2017-4330 30420470 10.1542/peds.2017-4330

[57] Peters JL, Sutton AJ, Jones DR, Abrams KR, Rushton L (2007) Performance of the trim and fill method in the presence of publication bias and between-study heterogeneity. Stat Med 26(25):4544–4562. 10.1002/sim.2889 17476644 10.1002/sim.2889

[58] Pinheiro KA, Pinheiro RT, Silva RA, Coelho FM, Quevedo Lde Á, Godoy RV, Jansen K, Lessa Horta B, Oses JP (2011) Chronicity and severity of maternal postpartum depression and infant sleep disorders: a population-based cohort study in southern Brazil. Infant Behav Dev 34(2):371–373. 10.1016/j.infbeh.2010.12.006 21211848 10.1016/j.infbeh.2010.12.006

[59] Räikkönen K, Seckl JR, Pesonen AK, Simons A, Van den Bergh BR (2011) Stress, glucocorticoids and liquorice in human pregnancy: programmers of the offspring brain. Stress 14(6):590–603. 10.3109/10253890.2011.602147 21875300 10.3109/10253890.2011.602147

[60] Sadeh A, Anders TF (1993) Infant sleep problems: origins, assessment, interventions. Infant Ment Health J 14(1):17–34

[61] Sadeh A, Tikotzky L, Kahn M (2014) Sleep in infancy and childhood: implications for emotional and behavioral difficulties in adolescence and beyond. Curr Opin Psychiatry 27(6):453–459. 10.1097/yco.0000000000000109 25247458 10.1097/YCO.0000000000000109

[62] Sadeh A, Tikotzky L, Scher A (2010) Parenting and infant sleep. Sleep Med Rev 14(2):89–96. 10.1016/j.smrv.2009.05.003 19631566 10.1016/j.smrv.2009.05.003

[63] Scher A, Zukerman S, Epstein R (2005) Persistent night waking and settling difficulties across the first year: early precursors of later behavioural problems? J Reprod Infant Psychol 23(1):77–88. 10.1080/02646830512331330929

[64] Simard V, Chevalier V, Bédard M-M (2017) Sleep and attachment in early childhood: a series of meta-analyses. Attach Hum Dev 19(3):298–321. 10.1080/14616734.2017.1293703 28277095 10.1080/14616734.2017.1293703

[65] Simard V, Lara-Carrasco J, Paquette T, Nielsen T (2011) Breastfeeding, maternal depressive mood and room sharing as predictors of sleep fragmentation in 12-week-old infants: a longitudinal study. Early Child Dev Care 181(8):1063–1077. 10.1080/03004430.2010.513434

[66] Simcock G, Cobham VE, Laplante DP, Elgbeili G, Gruber R, Kildea S, King S (2019) A cross-lagged panel analysis of children’s sleep, attention, and mood in a prenatally stressed cohort: the QF2011 Queensland flood study. J Affect Disord 255:96–104. 10.1016/j.jad.2019.05.041 31150945 10.1016/j.jad.2019.05.041

[67] Tesler N, Gerstenberg M, Huber R (2013) Developmental changes in sleep and their relationships to psychiatric illnesses. Curr Opin Psychiatry 26(6):572–579. 10.1097/YCO.0b013e328365a335 24060918 10.1097/YCO.0b013e328365a335

[68] Teti DM, Crosby B (2012) Maternal depressive symptoms, dysfunctional cognitions, and infant night waking: the role of maternal nighttime behavior. Child Dev 83(3):939–953. 10.1111/j.1467-8624.2012.01760.x 22506917 10.1111/j.1467-8624.2012.01760.x

[69] Tikotzky L, Volkovich E, Meiri G (2021) Maternal emotional distress and infant sleep: A longitudinal study from pregnancy through 18 months. Dev Psychol 57(7):1111–1123. 10.1037/dev0001081 34435826 10.1037/dev0001081

[70] Tuladhar CT, Schwartz S, St John AM, Meyer JS, Tarullo AR (2021) Infant diurnal cortisol predicts sleep. J Sleep Res 30(6):e13357. 10.1111/jsr.13357 33870573 10.1111/jsr.13357

[71] Wake M, Morton-Allen E, Poulakis Z, Hiscock H, Gallagher S, Oberklaid F (2006) Prevalence, stability, and outcomes of cry-fuss and sleep problems in the first 2 years of life: prospective community-based study. Pediatrics 117(3):836–842. 10.1542/peds.2005-0775 16510665 10.1542/peds.2005-0775

[72] Warren SL, Howe G, Simmens SJ, Dahl RE (2006) Maternal depressive symptoms and child sleep: models of mutual influence over time. Dev Psychopathol 18(1):1–16. 10.1017/s0954579406060019 16478549 10.1017/S0954579406060019

[73] Ystrom E, Hysing M, Torgersen L, Ystrom H, Reichborn-Kjennerud T, Sivertsen B (2017) Maternal symptoms of anxiety and depression and child nocturnal awakenings at 6 and 18 months. J Pediatr Psychol 42(10):1156–1164. 10.1093/jpepsy/jsx066 28369506 10.1093/jpepsy/jsx066 PMC5896619

[74] Ystrom H, Nilsen W, Hysing M, Sivertsen B, Ystrom E (2017) Sleep problems in preschoolers and maternal depressive symptoms: An evaluation of mother- and child-driven effects. Dev Psychol 53(12):2261–2272. 10.1037/dev0000402 28933879 10.1037/dev0000402

[75] Zijlmans MA, Riksen-Walraven JM, de Weerth C (2015) Associations between maternal prenatal cortisol concentrations and child outcomes: a systematic review. Neurosci Biobehav Rev 53:1–24. 10.1016/j.neubiorev.2015.02.015 25795521 10.1016/j.neubiorev.2015.02.015

